# Supplementary material for: The epidemiology of falls in Portugal: An analysis of hospital admission data
Source: PLoS One. 2021 Dec 22;16(12):e0261456. doi: 10.1371/journal.pone.0261456 (PMC8694436; doi:10.1371/journal.pone.0261456)
Supplement: S1 Table — (DOCX) [file pone.0261456.s001.docx]

S1 Table. Distribution of rates of falls (per 100,000 population at risk) by sex based on patients and admissions related to falls, between 2010 and 2018, in the Portuguese population.

| **Year** | **Population** | | **Number of admissions** | | **Rate (based on number of admissions)** | | **Number of patients** | | **Rate (based on number of patients)** | |
| --- | --- | --- | --- | --- | --- | --- | --- | --- | --- | --- |
|  | **M** | **F** | **M** | **F** | **M** | **F** | **M** | **F** | **M** | **F** |
| 2010 | 5,053,543 | 5,519,178 | 16,743 | 21,545 | 331.3 | 390.4 | 14,492 | 19,344 | 286.8 | 350.5 |
| 2011 | 5,030,437 | 5,511,961 | 18,762 | 24,830 | 373.0 | 450.5 | 15,389 | 21,126 | 305.9 | 383.3 |
| 2012 | 4,995,697 | 5,491,592 | 17,503 | 24,073 | 350.4 | 438.4 | 15,247 | 21,496 | 305.2 | 391.4 |
| 2013 | 4,958,020 | 5,469,281 | 18,862 | 25,311 | 380.4 | 462.8 | 16,669 | 22,788 | 336.2 | 416.7 |
| 2014 | 4,923,666 | 5,451,156 | 17,945 | 25,843 | 364.5 | 474.1 | 16,031 | 23,329 | 325.6 | 428.0 |
| 2015 | 4,901,509 | 5,439,821 | 18,557 | 26,560 | 378.6 | 488.3 | 16,706 | 24,339 | 340.8 | 447.4 |
| 2016 | 4,882,456 | 5,427,117 | 19,194 | 27,998 | 393.1 | 515.9 | 17,548 | 26,110 | 359.4 | 481.1 |
| 2017 | 4,867,692 | 5,423,335 | 17,100 | 24,942 | 351.3 | 459.9 | 15,816 | 23,455 | 324.9 | 432.5 |
| 2018 | 4,852,366 | 5,424,251 | 14,947 | 22,298 | 308.0 | 411.1 | 13,839 | 21,012 | 285.2 | 387.4 |

F: Females; M: Males
