# Supplementary material for: The epidemiology of falls in Portugal: An analysis of hospital admission data
Source: PLoS One. 2021 Dec 22;16(12):e0261456. doi: 10.1371/journal.pone.0261456 (PMC8694436; doi:10.1371/journal.pone.0261456)
Supplement: S2 Table — (DOCX) [file pone.0261456.s002.docx]

S2 Table. Distribution of number of patients and inpatient admissions related to falls, by age groups, between 2010 and 2018, in the Portuguese population.

| **Age groups** | **2010** | | **2011** | | **2012** | | **2013** | | **2014** | | **2015** | | **2016** | | **2017** | | **2018** | | **Total** | |
| --- | --- | --- | --- | --- | --- | --- | --- | --- | --- | --- | --- | --- | --- | --- | --- | --- | --- | --- | --- | --- |
|  | **A** | **P** | **A** | **P** | **A** | **P** | **A** | **P** | **A** | **P** | **A** | **P** | **A** | **P** | **A** | **P** | **A** | **P** | **A** | **P** |
| 0-5 | 717 | 564 | 788 | 668 | 686 | 599 | 642 | 569 | 596 | 550 | 651 | 592 | 572 | 525 | 528 | 493 | 425 | 389 | 5605 | 4949 |
| 5-10 | 906 | 825 | 959 | 842 | 863 | 788 | 850 | 762 | 829 | 763 | 819 | 755 | 809 | 754 | 717 | 671 | 564 | 526 | 7316 | 6686 |
| 10-15 | 890 | 808 | 897 | 801 | 866 | 792 | 883 | 799 | 856 | 789 | 795 | 727 | 853 | 810 | 650 | 611 | 508 | 484 | 7198 | 6621 |
| 15-20 | 622 | 550 | 720 | 613 | 661 | 585 | 656 | 596 | 652 | 580 | 630 | 581 | 640 | 597 | 455 | 430 | 381 | 358 | 5417 | 4890 |
| 20-25 | 625 | 531 | 697 | 571 | 602 | 518 | 559 | 490 | 541 | 473 | 511 | 459 | 528 | 474 | 446 | 394 | 316 | 284 | 4825 | 4194 |
| 25-30 | 695 | 620 | 798 | 651 | 665 | 568 | 638 | 553 | 542 | 473 | 567 | 505 | 597 | 555 | 465 | 415 | 355 | 314 | 5322 | 4654 |
| 30-35 | 906 | 791 | 956 | 806 | 844 | 692 | 757 | 666 | 719 | 641 | 672 | 594 | 726 | 663 | 567 | 515 | 445 | 392 | 6592 | 5760 |
| 35-40 | 1138 | 999 | 1241 | 1017 | 1007 | 875 | 1087 | 955 | 995 | 877 | 964 | 864 | 936 | 870 | 678 | 627 | 596 | 546 | 8642 | 7630 |
| 40-45 | 1211 | 1040 | 1376 | 1123 | 1211 | 1039 | 1209 | 1084 | 1132 | 1020 | 1176 | 1070 | 1232 | 1133 | 1014 | 943 | 842 | 768 | 10403 | 9220 |
| 45-50 | 1441 | 1257 | 1683 | 1384 | 1483 | 1295 | 1509 | 1310 | 1402 | 1265 | 1438 | 1293 | 1498 | 1397 | 1243 | 1146 | 1022 | 959 | 12719 | 11306 |
| 50-55 | 1664 | 1478 | 2013 | 1653 | 1721 | 1498 | 1870 | 1674 | 1789 | 1609 | 1774 | 1614 | 2007 | 1837 | 1633 | 1534 | 1416 | 1324 | 15887 | 14221 |
| 55-60 | 1930 | 1691 | 2154 | 1785 | 2020 | 1774 | 2288 | 2021 | 2208 | 1970 | 2202 | 2009 | 2345 | 2164 | 2146 | 1973 | 1787 | 1668 | 19080 | 17055 |
| 60-65 | 2217 | 1916 | 2358 | 1926 | 2429 | 2089 | 2690 | 2388 | 2537 | 2262 | 2595 | 2336 | 2799 | 2579 | 2420 | 2246 | 2161 | 2005 | 22206 | 19747 |
| 65-70 | 2573 | 2270 | 3013 | 2459 | 2903 | 2507 | 3166 | 2767 | 3058 | 2695 | 3185 | 2839 | 3345 | 3048 | 2993 | 2771 | 2735 | 2559 | 26971 | 23915 |
| 70-75 | 3628 | 3193 | 3977 | 3296 | 3601 | 3168 | 3981 | 3508 | 3774 | 3375 | 3958 | 3581 | 4125 | 3781 | 3837 | 3573 | 3446 | 3182 | 34327 | 30657 |
| 75-80 | 4755 | 4207 | 5390 | 4514 | 5300 | 4749 | 5438 | 4877 | 5666 | 5093 | 5936 | 5406 | 5820 | 5385 | 5226 | 4880 | 4475 | 4209 | 48006 | 43320 |
| 80-85 | 5330 | 4784 | 6273 | 5337 | 6185 | 5576 | 6892 | 6231 | 6793 | 6129 | 6970 | 6365 | 7458 | 6930 | 6756 | 6366 | 6100 | 5745 | 58757 | 53463 |
| ≥85 | 7040 | 6356 | 8299 | 7124 | 8531 | 7679 | 9058 | 8265 | 9699 | 8860 | 10273 | 9519 | 10903 | 10219 | 10268 | 9740 | 9671 | 9207 | 83742 | 76969 |

A: Admissions; P: Patients
